# Evolutionary patterns of volatile terpene emissions across 202 tropical tree species

**DOI:** 10.1002/ece3.1810

**Published:** 2016-03-22

**Authors:** Elodie A. Courtois, Kyle G. Dexter, Charles Eliot Timothy Paine, Didier Stien, Julien Engel, Christopher Baraloto, Jérôme Chave

**Affiliations:** ^1^ CNRS GuyaneUSR 34562, Avenue Gustave Charlery97300CayenneFrance; ^2^ Laboratoire Evolution et Diversité BiologiqueUMR 5174 CNRS/Université Paul Sabatier118, route de Narbonne31062ToulouseFrance; ^3^ CNRS, UMR EcofogUniversité Antilles GuyaneBP 70997387Kourou CedexFrance; ^4^ School of GeoSciencesUniversity of EdinburghEdinburghEH9 3FFUK; ^5^ Royal Botanic Garden Edinburgh20a Inverleith RowEdinburghEH3 5LRUK; ^6^ Biological and Environmental SciencesUniversity of StirlingStirlingFK9 4LAUK; ^7^ Laboratoire de Biodiversité et Biotechnologies Microbiennes (LBBM)Observatoire OcéanologiqueSorbonne Universités, UPMC Univ Paris 06, CNRS66650Banyuls‐sur‐merFrance; ^8^ INRAUMR EcofogBP 70197387Kourou CedexFrance; ^9^ International Center for Tropical BotanyDepartment of Biological SciencesFlorida International UniversityMiamiFlorida33199

**Keywords:** Chemical defense, French Guiana, herbivory, secondary metabolites, tropical forest

## Abstract

Plant responses to natural enemies include formation of secondary metabolites acting as direct or indirect defenses. Volatile terpenes represent one of the most diverse groups of secondary metabolites. We aimed to explore evolutionary patterns of volatile terpene emission. We measured the composition of damage‐induced volatile terpenes from 202 Amazonian tree species, spanning the angiosperm phylogeny. Volatile terpenes were extracted with solid‐phase micro extraction and desorbed in a gas chromatography–mass spectrometry for compound identification. The chemical diversity of the terpene blend showed a strong phylogenetic signal as closely related species emitted a similar number of compounds. Closely related species also tended to have compositionally similar blends, although this relationship was weak. Meanwhile, the ability to emit a given compound showed no significant phylogenetic signal for 200 of 286 compounds, indicating a high rate of diversification in terpene synthesis and/or great variability in their expression. Three lineages (Magnoliales, Laurales, and Sapindales) showed exceptionally high rates of terpene diversification. Of the 70 compounds found in >10% of their species, 69 displayed significant correlated evolution with at least one other compound. These results provide insights into the complex evolutionary history of volatile terpenes in angiosperms, while highlighting the need for further research into this important class of compounds.

## Introduction

The interactions of plants with pollinators, herbivores, and pathogens are mediated by the chemical compounds they emit (Hartmann 2007). Such compounds have been historically defined as secondary metabolites, that is chemical compounds that are not used directly for growth or reproduction (Fraenkel 1959). They are characterized in plants by a large structural diversity, with over 200,000 compounds identified to date, with many more expected to be discovered (Hartmann 2007). Terpenes represent one of the largest groups of secondary metabolites, with approximately 25,000 structures reported to date (Gershenzon and Dudareva 2007). Some terpenes (monoterpenes and sesquiterpenes) are volatile and can be stored in specialized organs in leaves, stems, and trunks and released while others are synthesized de novo after wounding (Fineschi et al. 2013; Lange 2015). The main role of these compounds is to defend against pathogens and herbivores (Unsicker et al. 2009) by direct toxicity, repulsion of herbivores, or the attraction of herbivores' enemies (Heil 2008). The blend of volatile compounds emitted by a given plant species can be highly complex, including up to 200 different compounds (Dicke and van Loon 2000).

A family of enzymes named terpene synthases (TPSs) is responsible for the synthesis of isoprene, monoterpenes, and sesquiterpenes from two isomeric 5‐carbon precursor “building blocks” (Dudareva et al. 2004; Chen et al. 2011). TPS diversity appears to originate from repeated duplication and subsequent divergence of an ancestral TPS gene family important in primary metabolism (Bohlmann et al. 1998). Minor changes in the structure of TPSs can lead to major changes in product profiles, indicating that the rich terpenoid genetic diversity could constitute a means of rapid evolutionary adaptation to novel biotic interactions (Memari et al. 2013). The genomic architecture of TPS expression is complex (Chen et al. 2011) and the expression of a particular TPS usually results in multiple products (Bohlmann et al. 1998). Biosynthetic pathways for terpene production are present in the extant lineages that are most closely related to flowering plants (Chen et al. 2011), so it is reasonable to assume that they arose early in the evolution of land plants (Croteau et al. 2000; Wink 2003).

Patterns and processes of terpene diversification in plants remain subject to debate (Hanson et al. 1999; Harley et al. 1999; Fineschi et al. 2013). There may be several selective advantages to maintaining a large diversity of volatile terpenes in individuals (Gershenzon and Dudareva 2007). First, in a world where multiple natural enemies are encountered, it could provide a more potent defense and biological activity against a spectrum of aggressors (Firn and Jones 2006). There is evidence, for example, that different monoterpenes in thyme (*Thymus*) deter different species of herbivores and inhibit different pathogens (Linhart and Thompson 1999), suggesting that a more diverse blend of compounds can protect against a broader range of enemies. Second, the blend of volatiles emitted by a plant may be a more efficient deterrent than the sum of its parts (Gershenzon and Dudareva 2007), as several studies point out that mixtures are more active than pure compounds (Lin et al. 1987). It is therefore expected that species or lineages that invest more in this mechanism of defense should display both a higher diversity in the emitted blend at the species level and a higher rate of diversification of compounds in the lineage.

Tropical tree species produce a large diversity of volatile terpenes, but the diversity of compounds can differ markedly among genera and families (Courtois et al. 2009). There has been considerable recent interest in exploring how sympatric species within the same assemblage partition ecological resources and functional trait space, as one of the major mechanisms of species coexistence may be niche partitioning (Cavender‐Bares et al. 2009). The great diversity of moist tropical forests has motivated much of this recent research. Most recent empirical studies of niche partitioning in tropical trees have focused on leaf‐level and trunk‐level traits related to resource acquisition (Kraft and Ackerly 2010; Baraloto et al. 2012). However, the coexistence of diverse assemblages of tropical trees may be attributable to negative density‐dependent effects mediated by natural enemies such as pathogens and herbivores (Harms et al.*,* 2000, Wright 2002; Novotny et al. 2010), which would involve partitioning of defense niches, rather than resource‐use niches. Even so, very few studies have explored the partitioning of defense niches, particularly chemical defense niches (but see Kursar et al. 2009).

In this study, we combine an original dataset of volatile terpene composition across 202 tropical tree species with a phylogenetic hypothesis for those species to explore patterns of volatile terpene evolution in tropical trees. More specifically, we test whether (1) the diversity (i.e., total number) and composition of compounds shows significant phylogenetic signal, *that is* whether closely related species tend to share a more similar blend of compounds in terms of the total number of emitted compounds and identity of compounds, (2) individual compounds display phylogenetic signal, (3) the pattern of diversification of compounds is the same in different lineages, and (4) compounds display significant correlated evolution with each other across the phylogeny.

## Materials and Methods

### Collection of plant tissue

Field sampling was conducted in nine 1‐ha permanent tree plots that span coastal French Guiana (Baraloto et al. 2012). In each plot, all trees >10 cm diameter at breast height (d.b.h.) were mapped, measured, and collected for herbarium vouchers (deposited at the Cayenne herbarium) and silica gel‐dried leaf DNA vouchers. All plants were subsequently identified by taxonomic specialists (Baraloto et al. 2012). For analysis of VOC composition, we selected 202 species across 172 genera, representing nearly half of the 380 tree genera present in the Guiana Shield (de Granville 1988) and spanning the breadth of the angiosperm phylogenetic tree. A previous study on a subset of the same species revealed low to absent intraspecific variation in volatile terpene presence/absence (Courtois et al. 2009). Of the 44 species with more than three sampled individuals, only 8 displayed an ambiguous pattern in an unsupervised clustering analysis (18%; see fig. 3 of Courtois et al. 2009), with no hard conflicts. For this study, we therefore decided to include a single individual per species, which allowed us to include rare species and span a broader range of chemical diversity.

For each sampled tree, we collected ~20 mg of fresh leaf tissue and a 1‐cm² piece of bark at 1 m above the ground using a leather punch. Each sample was immediately placed in a glass vial (10 mL) and sealed with a screw cap containing a Teflon‐lined septum (Varian Instruments, Sunnyvale, CA, USA). Sealed vials were maintained at −4°C in a portable field freezer until arrival at the laboratory, where they were stored at −20°C until analysis.

### Phylogenetic reconstruction

We pruned the phylogenetic tree described in Baraloto et al. (2012) to species for which VOCs were sampled. Full details regarding laboratory protocols and phylogeny construction are provided by Baraloto et al. (2012), but we provide the main details here. Up to 30 mg of dry leaf tissue was ground for 2 min in a TissueLyser mixer‐mill disruptor (Qiagen, Venlo, Netherlands) using tungsten beads. Lysis incubation using CTAB 1% PVP buffer was carried out at 65°C for ≥2 h for cambium tissue and 1 h for leaf tissue. Total DNA extraction was performed using a Biosprint 15 workstation (Qiagen) following the manufacturer's protocols. PCR amplification and sequencing of sections of the *rbc*L and *mat*K chloroplast genes was then conducted. A total of 606 *rbcL* sequences (1320 bp), and 244 *matK* sequences (891 bp) were obtained, with at least one sequence per genus for *matK*. These sequences were deposited in GenBank/EBI (accession numbers JQ625717–JQ626579).

A dated phylogenetic hypothesis was constructed as follows. First a maximum‐likelihood phylogenetic tree was generated using RAxML v7.0.1 software (Stamatakis et al. 2008), and this tree was made ultrametric using Sanderson's (1997) nonparametric rate smooth (NPRS) method. The ultrametric tree was then used as the starting tree in BEAST v1.5.3 (Drummond & Rambaut 2007). We implemented an uncorrelated relaxed molecular clock, which draws substitution rates from a log‐normal distribution independently for each branch in the phylogenetic tree. In order to calibrate the phylogenetic tree, we used fossil ages to place constraints on divergence times for minimum crown age at nine nodes spread across the phylogeny (see Appendix S2 in Baraloto et al. 2012). We ensured that the runs had converged after 30 million generations. Using the ultrametric tree, we calculated the phylogenetic distance between all pairs of species as the total branch length separating them.

### Chemical analysis

Volatiles were extracted with a solid‐phase micro extraction (SPME) fiber of the type PDMS/DVB (polydimethylsiloxane/divinylbenzene) 65 *μ*m (Supelco, Bellefonte, PA). Fibers were conditioned before the first use for 30 min at 250°C, following instructions from the manufacturer. Before extraction, the glass vials containing the tissue samples were maintained at room temperature for at least 1 h. The SPME fiber was placed into the vial with the tissue sample (bark or leaf) for 5–60 min at ambient temperature (25°C, exposure time was optimized for each species to maximize the extraction without saturating the analytical column). The fiber was inserted immediately into the 250°C inlet of a Varian 3800 Gas Chromatograph (GC) fitted with a Saturn 2000 ion‐trap Mass Spectrometer (MS; Varian Instruments, Sunnyvale, CA). The GC was run with a nonpolar Varian DB‐5 column (30 m × 0.25 mm ID, 0.25 *μ*m film), and helium was the carrier gas at a constant flow of 1 mL/min. The oven temperature program of the GC started at 50°C, with 6°C/min temperature increase up to 140°C, and then with 4°C/min increments up to 160°C. This temperature was held for 1 min and increased finally to 200°C at 10°C/min. The MS was operated in electron impact (EI) mode at 70 eV, with a scan range of 30–450 m/z. After each analysis, the fibers were cleaned in the injector port for 10 min at 250°C, and each fiber was reused no more than 100 times. Control analyses (blanks) were performed every ten analyses to check for contamination of the fiber.

We used a subsample of species (*Protium* sp. Burseraceae, *Inga* sp. Mimosaceae, *Guarea* sp. Meliaceae, and *Spondias mombin* Anacardiaceae) to confirm that storage of leaf or bark tissue samples at −20°C prior to analysis did not result in drastic changes in the composition of volatiles (e.g., due to frost damage). For further details on chemical protocols, see Courtois et al. (2009).

For chemical composition, we inferred the presence/absence of chemicals from the chromatograms using a novel statistical approach implemented in the MSeasy package (Nicolè et al. 2012) in the R Statistical Environment (http://cran.r-project.org/), which is both more efficient and more accurate than visual classification procedures when conducting a large number of analyses. This method is based on the mass spectrum of each detected molecule and the corresponding Kováts Retention Index (Kováts 1958). Molecules were identified based on the comparison of mass spectra with standards or with the NIST 98 MS library, the ADAMS library (Adams 2007), and with RI reported in the literature. Unidentified molecules were defined as morphomolecules characterized by their mass spectrum and their RI and assigned to a class of compounds (monoterpenes or sesquiterpenes) based on ion fragmentation. Overlapping peaks were checked manually. We defined the chemical composition for each individual tree as the union of all compounds found in the leaves or bark.

### Evolutionary analyses

#### Measures of phylogenetic signal of the overall blend of compounds

The diversity (number of compounds of the emitted blend) can be an important component of efficiency of defense. We estimated phylogenetic signal for the total number of compounds emitted by each species using Pagel's lambda (Pagel 1999). This metric varies from 0 to 1, with 0 indicating no phylogenetic signal and 1 indicating high phylogenetic signal, or a perfect correlation of a trait with phylogeny under a Brownian motion model. We used likelihood ratio tests to determine whether the estimate of phylogenetic signal was significantly greater than zero. Moreover, we assessed whether closely related species tend to share a more similar blend of compounds using a Mantel test of the correlation between chemical dissimilarity and species pairwise phylogenetic distances. Chemical dissimilarity was measured by the pairwise Manhattan distance and the number of compounds that are present in one species but not in the other (see Legendre and Cáceres 2013).

#### Measures of phylogenetic signal of individual compounds

We tested for phylogenetic signal for each compound found in more than one species using the D metric for binary traits developed by Fritz and Purvis (2010). The D statistic is equal to 1 if the observed binary trait has a phylogenetically random distribution across the tips of the phylogeny and to 0 if the observed trait is phylogenetically clustered as if it had evolved by Brownian motion (null expectation). Using 1000 permutations, a *P*‐value is generated to test whether the observed value of D is significantly different from 1 (a random distribution) or 0 (the null expectation).

#### Character reconstruction to estimate pattern of diversification in lineages

We used two approaches to reconstruct the evolutionary history of all volatile terpenes found in at least two species. For comparability with previous studies, we used maximum parsimony reconstruction, hereafter MPR (Swofford and Maddison 1987). This approach unrealistically assumes that only one change can happen on a single phylogenetic branch, no matter how long that branch is, and it underestimates uncertainty in ancestral state assignments (Bollback 2006). We therefore additionally implemented stochastic character mapping, hereafter SCM (Nielsen 2002; Huelsenbeck et al. 2003). SCM uses a probabilistic approach to reconstruct character states based on a specified model of character evolution and allows characters to change more than once along branches in a phylogenetic tree (Huelsenbeck et al. 2003). For both approaches, we determined the minimum number of gains of each compound, either across all maximally parsimonious reconstructions, in the case of MPR, or across 100 simulations, in the case of SCM. We also determined the state of compounds at the root node and considered a compound as ancestral of all taxa if it was reconstructed as unambiguously present under MPR or as present with a probability greater than 0.9 under SCM (i.e., reconstructed as present in >90% of the simulations). For two‐state character reconstruction using SCM, two models can be used. In the ARD (all rates different) model, the rate of change from one state to the other can be different from the reverse change; while under ER (equal rate) model, the two rates are constrained to be equal. For each compound, we compared the likelihood of these two models with a likelihood ratio test (see below for details) and used the most likely model for simulations (Table S1).

#### Correlated evolution analysis

We conducted analyses to determine the degree of correlated evolution among compounds. For the compounds that were found in more than 10% of species (i.e., over 20 species), we tested for correlated evolution using the BayesDiscrete module implemented in the BayesTraits software (Pagel and Meade 2006). Correlated evolution is tested by comparing the fit (log‐likelihood) of two models: one model with independent evolution of the two traits and one with correlated evolution of the traits. In order to test which model is a better fit to the data, we computed a likelihood ratio (LR) as LR = 2 × (ln_ML2_ ‐ ln_ML1_, where ln_ML1_ is the log‐likelihood of the independent model and ln_ML2_ is the log‐likelihood of the correlated model. The LR follows a chi‐squared distribution with four degrees of freedom. When the log‐likelihood of the correlated evolution model is significantly greater than the log‐likelihood of the independent model, there is support for correlated evolution of the two traits.

Evolutionary analyses were conducted in the R Statistical Environment (R Core Development Team) using the following packages: caper (Orme 2012), ape (Paradis et al. 2004), phytools (Revell 2012), and phangorn (Schliep 2011), except for the correlated evolution analyses, which were conducted with the BayesTraits software (Pagel and Meade 2006).

## Results

All 202 species assessed emitted at least one volatile terpene compound. Yet, we observed high variation in the diversity of emitted volatile terpenes, with species emitting between 1 and 30 monoterpenes (9.2 ± 4.5 SD) and between 0 and 59 sesquiterpenes (21.5 ± 14.3 SD). Overall, 376 terpenes were detected across the 202 species, 88 being monoterpenes and 288 being sesquiterpenes. Among these, 59 monoterpenes (67%) and 114 sesquiterpenes (40%) could be identified based on the comparison of mass spectra with standards or with libraries and retention indices reported in the literature. The proportion of compounds found in a single species (*i.e*., singletons) was twice as high for monoterpenes (39%; 34 of 88 compounds) as for sesquiterpenes (19%; 56 of 288 compounds).

**Figure 1:**
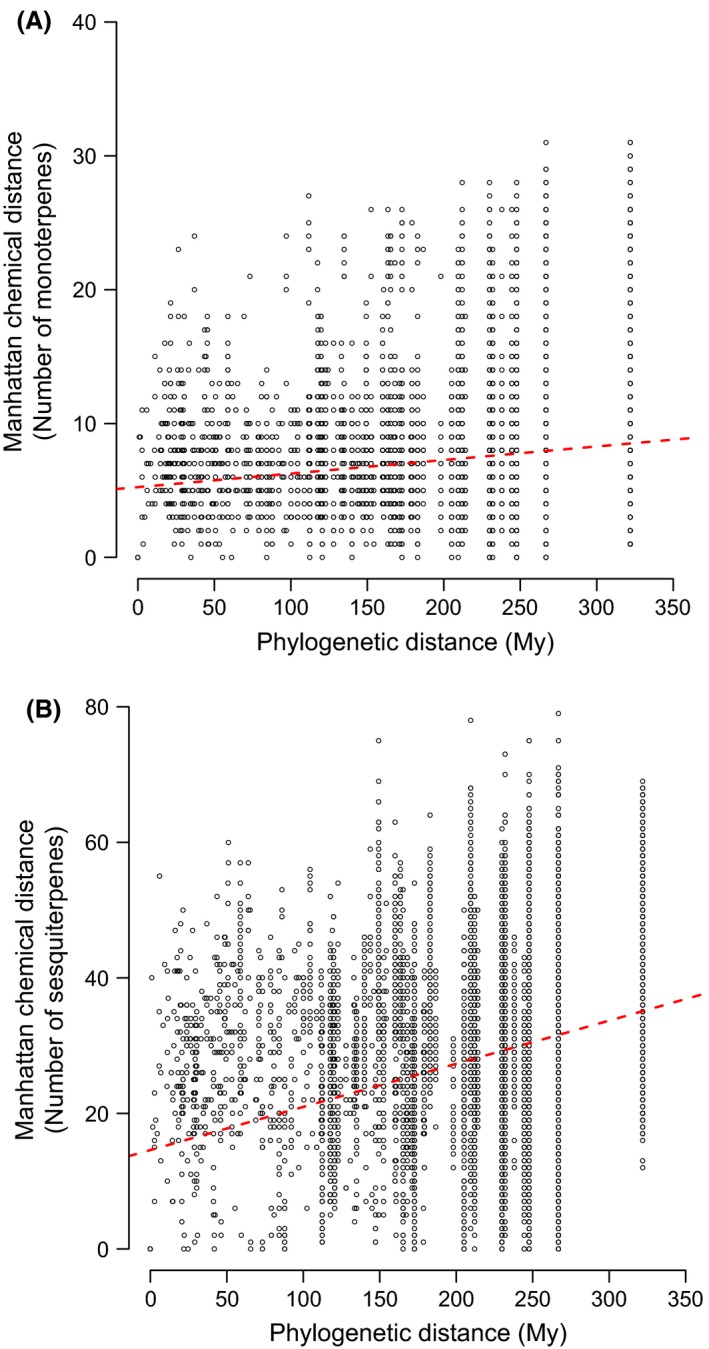
Representation of chemical dissimilarity for (A) monoterpenes and (B) sesquiterpenes vs. phylogenetic dissimilarity. Each point represents a pairwise comparison of two species characterized by their Manhattan chemical dissimilarity (number of compounds that are present in one species but not in the other) and their phylogenetic dissimilarity (computed using branch length, distance in My among the two species). The dotted red lines represent the regression line between the two values.

The total number of compounds found in each species exhibited high and significant phylogenetic signal (*λ *= 0.65, *P* < 0.01), although this pattern was stronger for sesquiterpenes (*λ *= 0.71, *P* < 0.01) than for monoterpenes (*λ *= 0.14, *P* = 0.04). The composition of the overall blend of compounds showed weak but significant phylogenetic signal (Mantel *r* = 0.15, *P* < 0.001). As with the total number of compounds, the pattern with respect to sesquiterpenes was stronger (sesquiterpenes: Mantel *r* = 0.26, *P* < 0.001; monoterpenes: Mantel *r* = 0.11, *P* < 0.001, Fig. 1).

When considering each compound separately, 75 of the 232 sesquiterpenes that were found in more than one species (32%, Table S1) showed significant phylogenetic signal, whereas only 11 of the 54 monoterpenes found in more than one species (20%, Table S1) showed significant phylogenetic signal.

Of the 286 compounds emitted by more than one species, only seven compounds, including five monoterpenes (*α*‐pinene, limonene, *β*‐pinene, camphene, and *p*‐cymene) and two sesquiterpenes (*β*‐caryophyllene and *α*‐copaene), were reconstructed as present at the root of the phylogeny by both methods (SCM – stochastic character mapping and MPR – maximum parsimony reconstruction, Table S1). An additional four compounds (2 sesquiterpenes; *α*‐cubebene and *δ*‐cadinene, and 2 monoterpenes; 3‐carene and *α*‐thujene) were inferred to be present at the root of the phylogeny using MPR (Table S1). Under both methods, nearly all compounds (284 of 286 using SCM and 280 using MPR) had a minimum number of gains greater than 1 (Table S1).

**Figure 2:**
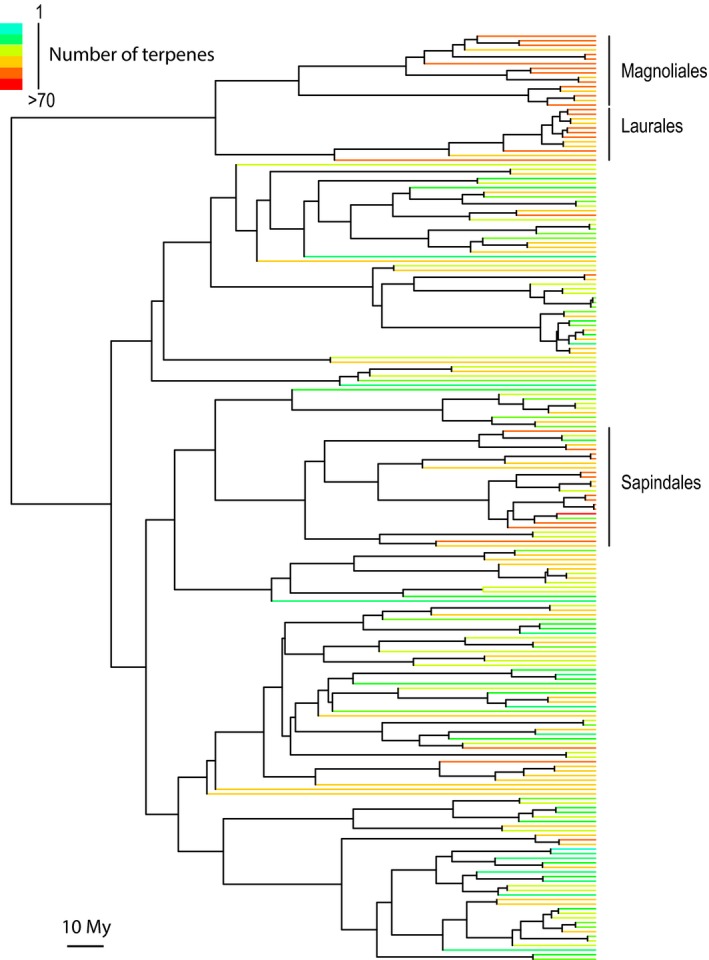
Phylogenetic tree described in Baraloto et al. (2012) of the 202 studied species with each terminal branches colored by the number of terpenes found in the descendent node from green (low number of compounds) to red (large number of compounds). Laurales, Magnoliales, and Sapindales are highlighted.

The mean number of monoterpenes and sesquiterpenes per species differed among taxonomic orders (Kruskal Wallis, *P* = 0.008 and *P* < 0.001, respectively). Species with the highest number of monoterpenes and sesquiterpenes belonged to the Magnoliales, Laurales and Sapindales (Table 1, Fig. 2). These three orders also contained the highest number of unique compounds (in addition to the Malphigiales; Table 1). Overall, 102 compounds among 376 were restricted to a single order and most of those (90 of 102, 88%) are singletons, that is found in only one species.

**Table 1:** Summary of the number of volatile compounds found in angiosperm orders, sorted by the mean number of terpenes per species. Note that the number of species examined differed among orders

	No. of species analyzed	Mean no. of monoterpenes per species	Mean no. of sesquiterpenes per species	Mean no. of terpenes per species	No. of unique monoterpenes	No. of unique sesquiterpenes	No. of unique terpenes
Brassicales	1	6	6	12	0	0	0
Lamiales	3	4.7	10.7	15.4	0	0	0
Santalales	5	8.2	11.4	19.6	0	0	0
Fabales	28	8	13.1	21.1	0	3	3
Rosales	8	8	14	22	0	2	2
Malvales	8	5.9	18	23.9	2	2	4
Ericales	20	8.8	16.5	25.3	3	4	7
Malpighiales	39	8.7	16.8	25.5	6	11	17
Aquifoliales	1	10	16	26	0	0	0
Celastrales	2	8	19	27	0	0	0
Gentianales	15	9.5	18.1	27.6	6	3	9
Myrtales	12	8.9	19	27.9	2	0	2
Garryales	3	11	20	31	0	2	2
Caryophyllales	2	7.5	24	31.5	0	0	0
Oxalidales	1	8	24	32	0	1	1
Sapindales	**26**	**10.7**	**33.8**	**44.5**	**4**	**16**	**20**
Laurales	**12**	**12.8**	**36.1**	**48.9**	**3**	**14**	**17**
Magnoliales	**16**	**11.7**	**40.3**	**52**	**10**	**8**	**18**

Bold values represent the three orders with the highest mean number of terpenes per species.

Of the 15 monoterpenes and the 55 sesquiterpenes that are found in more than 10% of the sampled species, all but one (*α*‐pinene) displayed significant correlated evolution with at least one other compound (Table S2 and Table S3). Moreover, 67% of these monoterpenes (10 of 15) and 45% of these sesquiterpenes (25 of 55) evolved in a correlated manner with at least five other compounds.

## Discussion

The emission of volatile terpenes represents an important plant defense strategy, but little is known about its evolutionary history in Neotropical lineages. In this study, we determined the composition of the emitted volatile terpene blend for 202 tropical tree species and examined patterns of compound evolution, diversification, and correlated evolution. We found that the diversity of the emitted blend (number of compounds) in individual species showed high phylogenetic signal and that the composition of the overall blend showed weak, albeit significant phylogenetic signal. Indeed, most of the individual compounds showed no significant phylogenetic signal. Certain lineages of plants showed significantly higher rates of compound diversification. Finally, we found a strong pattern of correlated evolution for common compounds (those found in >10% of species), with most such compounds displaying correlated evolution.

Significant phylogenetic signal of volatile blends could be explained by Brownian motion (i.e., random drift) evolution, phylogenetic conservatism, or evolutionary constraint in compound evolution (Losos 2008). Conversely, finding weaker phylogenetic signal than expected under Brownian motion suggests that diversifying selection for terpene composition may be occurring. The fact that monoterpenes displayed a weaker phylogenetic signal than sesquiterpenes might therefore suggest either a stronger diversifying selection in this group or a stronger constraining selective pressure on sesquiterpenes. Such results could also suggest, as have other studies, that the characteristic blend of volatiles emitted by wounded leaves might carry information about species identity (Courtois et al. 2009; Pearse et al. 2013) and phylogenetic relationships (Pearse and Hipp 2009). Most previous studies reporting phylogenetic conservatism in the blend of volatiles emitted by wounded leaves were based on within‐genus comparisons (Pearse et al. 2013). Our study generalizes these findings across a broader taxonomic and phylogenetic range.

When considering each compound independently, we found that few (20% of monoterpenes and 32% of sesquiterpenes) displayed phylogenetic signal. The few studies that have tested for phylogenetic signal of terpene emissions in a broad set of plant species have yielded the same result (e.g., Llusià et al. 2010, 2014). In these papers, the authors used more than one individual per species and highlighted the importance of both intraspecific variation and quantification of compounds, two aspects that are not taken into account in our study, yet they found similar results. The observed low phylogenetic signal could be explained by several characteristics of terpenes. It can reflect convergent evolution of compounds, known to be frequent for this class of molecules (Bohlmann et al. 1998). For example, limonene‐producing enzymes originated independently in gymnosperms and Lamiaceae (Bohlmann et al. 1998). However, it is likely that in some cases the pathway is not lost but merely “switched off,” *that is* plants retain the ability to switch them on again (Wink 2003). For example, in *Arabidopsis thaliana*, two ecotypes differ in their emission of (*E*)‐*β*‐ocimene, and this difference is due to a mutation that inactivates one allele of the (*E*)‐*β*‐ocimene‐producing synthase in one of the ecotypes (Huang et al. 2010). Moreover, it is known that VOC emissions can be regulated as elicitors released by herbivores (Mattiacci et al. 1994). Hence, the blend measured after the mechanical damage we executed in this study may not lead to the release of all the compounds that a given species can emit. Our sampling technique may therefore have led us to underestimate the actual ability of the species to express compounds. Thus, evolutionary and/or phenotypic changes in gene regulation may underlie our finding of nonsignificant phylogenetic signal for most compounds rather than high rates of compound evolution per se. Finally, we acknowledge that terpene synthases usually synthesize a spectrum of compounds and that a given compound can be synthesized by multiple synthases (Memari et al. 2013), which could be expressed in response to various environmental and biological cues (Penuelas and Llusia 2001). Therefore, even if the patterns we found here are strong, it is difficult to directly relate them to evolutionary changes in biosynthetic pathways.

We found that the diversity of the emitted blend showed strong phylogenetic signal and that diversification of volatile terpenes occurred preferentially in certain plant lineages. Three orders (Magnoliales, Laurales, and Sapindales) harbored species tending to display a large number of compounds. These same three orders also displayed the greatest number of compounds at the order level and the highest number of compounds that are restricted to a single order. This suggests that the capacity to generate novel compounds is not evenly distributed across the angiosperm phylogeny. From an ecological standpoint, a greater diversity of the emitted blend likely provides a better protection against a broader range of enemies (Gershenzon and Dudareva 2007). Such greater diversity in the emitted blend at species level and a higher rate of diversification of compounds in these lineages might therefore indicate that their defense strategy is focused on VOC synthesis and emissions. Other lineages may rely upon other means of defense against natural enemies (e.g., latex production, tough leaves).

We also found that at least two major events of diversification for volatile terpenes may have occurred in tropical trees: one corresponding to the Magnoliids (Magnoliales and Laurales), for which the stem age is estimated at 122–125 million years (Bell et al. 2010); and the other within the Sapindales for which divergence is suggested at ~ 70 million years (Magallón and Castillo 2009). Of course, we did not include all plant orders in our study, and similar diversification events may have occurred in other lineages that we have not sampled (e.g., those in the temperate zone).

Only seven compounds are reconstructed as unambiguously present at the root of the phylogeny (five monoterpenes and two sesquiterpenes). Given the high rate of changes in terpene expression, it is not surprising that we fail to unambiguously reconstruct ancestral states of most compounds deep in the phylogeny. Nonetheless, the fact that, even with this limitation, the unambiguous reconstruction of these seven compounds at the root of the phylogeny (with two different statistical approaches) could indicate that they are under more selective pressure to be retained than other compounds. In fact, among the five putatively ancestral monoterpenes, *α*‐pinene and limonene have been highlighted as compounds with the highest known repellent activity against insects (Nerio et al. 2010). They are also emitted by gymnosperms. Within the sesquiterpenes, the two compounds that can be considered as ancestral (*β*‐caryophyllene and *α*‐copaene) were already found to be frequent in tropical tree species (Courtois et al. 2009) and they are known to be implicated in direct and indirect defense against herbivores (Gols et al. 2008).

Most of the common compounds show evidence of correlated evolution, which is not unexpected given that most volatile terpenes are produced by only a few biosynthetic pathways (Chen et al. 2011). Some of the detected compounds may have no function but be present because they covary with a compound used in defense (Chen et al. 2011).

The emissions of terpenes have important effects on atmospheric chemistry and climate regulation (Fehsenfeld et al. 1992). Every species assessed in this study was found to emit terpenes, as found in other studies (Owen et al. 2001; Llusià et al. 2014). While we did not estimate the quantity of emitted terpenes, our study suggests that terpene emissions might be more important than previously thought in tropical trees and that forests differing in floristic composition will have important differences in volatile emissions. Our results therefore provide an important first step to parameterize models examining the effect of these compounds on atmospheric processes at broad spatial scales.

The high evolutionary variability of these compounds and the pervasive correlated evolution suggest a complex pattern of terpene biochemistry evolution. Nevertheless, this study suggests two major phases of diversification in volatile terpenes, one in the Magnoliids (Magnoliales and Laurales) and one within the Sapindales. This might indicate that volatile terpenes have been a major determinant of the defense strategy in these lineages (Becerra et al. 2009). These lineages may therefore be considered a good starting point for more mechanistic research into the regulation of terpene emission in tropical tree species.

Finally our study emphasizes the key role of chemistry in mediating biotic interactions across trophic levels in the tropics and suggests that the large diversity of compounds across coexisting species within tropical tree communities could be due, at least in part, to these biotic interactions.

## Conflict of Interest

None declared.

## Supporting information


**Table S1.** Measure of phylogenetic signal for each compound using the D metric for binary traits with the *P*‐value for departure from a random distribution and results from the SIMMAP simulation (ancestral state at the root of the tree, minimum number of gain and minimum number of losses).

**Table S2.** Coevolution of the 15 monoterpenes found in more than 20 species. The lower part of the matrix gives the LR (likelihood ratio) and the upper part of the matrix gives the *P*‐value of the test.

**Table S3.** Coevolution of the 55 sesquiterpenes found in more than 20 species. The lower part of the matrix gives the LR (likelihood ratio) and the upper part of the matrix gives the *P*‐value of the test.
